# How Is Global Warming Affecting Fruit Tree Blooming? “Flowering (Dormancy) Disorder” in Japanese Pear (*Pyrus pyrifolia*) as a Case Study

**DOI:** 10.3389/fpls.2021.787638

**Published:** 2022-02-10

**Authors:** Akiyoshi Tominaga, Akiko Ito, Toshihiko Sugiura, Hisayo Yamane

**Affiliations:** ^1^Faculty of Agriculture, Shizuoka University, Suruga, Japan; ^2^Institute of Fruit Tree and Tea Science, National Agriculture and Food Research Organization, Tsukuba, Japan; ^3^Graduate School of Agriculture, Kyoto University, Kyoto, Japan

**Keywords:** chilling requirement, cold accumulation, DAM, dormancy, floral bud maturation, warm temperature

## Abstract

Recent climate change has resulted in warmer temperatures. Warmer temperatures from autumn to spring has negatively affected dormancy progression, cold (de)acclimation, and cold tolerance in various temperate fruit trees. In Japan, a physiological disorder known as flowering disorder, which is an erratic flowering and bud break disorder, has recently emerged as a serious problem in the production of the pome fruit tree, Japanese (Asian) pear (*Pyrus pyrifolia* Nakai). Due to global warming, the annual temperature in Japan has risen markedly since the 1990s. Surveys of flowering disorder in field-grown and greenhouse-grown Japanese pear trees over several years have indicated that flowering disorder occurs in warmer years and cultivation conditions, and the risk of flowering disorder occurrence is higher at lower latitudes than at higher latitudes. Susceptibility to flowering disorder is linked to changes in the transcript levels of putative dormancy/flowering regulators such as *DORMANCY-ASSOCIATED MADS-box* (*DAM*) and *FLOWERING LOCUS T* (*FT*). On the basis of published studies, we conclude that autumn–winter warm temperatures cause flowering disorder through affecting cold acclimation, dormancy progression, and floral bud maturation. Additionally, warm conditions also decrease carbohydrate accumulation in shoots, leading to reduced tree vigor. We propose that all these physiological and metabolic changes due to the lack of chilling during the dormancy phase interact to cause flowering disorder in the spring. We also propose that the process of chilling exposure rather than the total amount of chilling may be important for the precise control of dormancy progression and robust blooming, which in turn suggests the necessity of re-evaluation of the characteristics of cultivar-dependent chilling requirement trait. A full understanding of the molecular and metabolic regulatory mechanisms of both dormancy completion (floral bud maturation) and dormancy break (release from the repression of bud break) will help to clarify the physiological basis of dormancy-related physiological disorder and also provide useful strategies to mitigate or overcome it under global warming.

## Introduction

**FIGURE 1:**
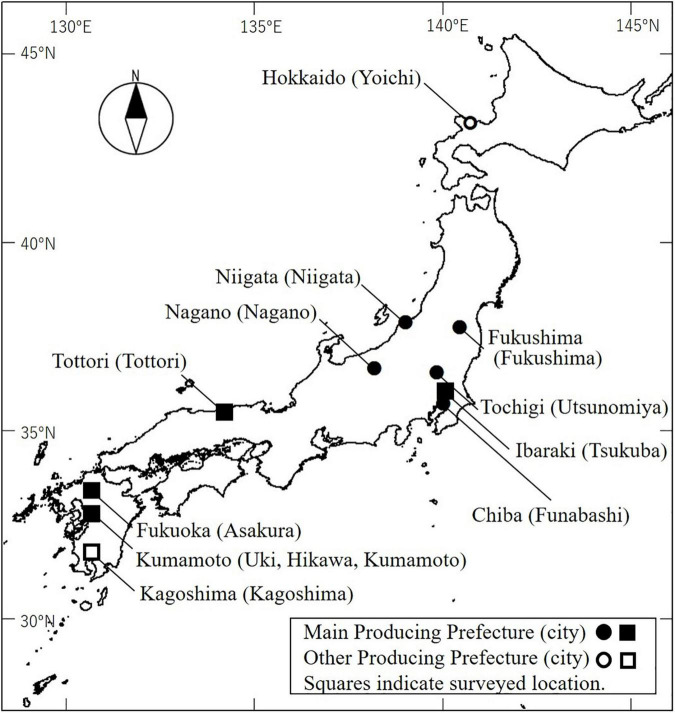
Locations of prefectures where Japanese pear is produced. Black markers indicate main production prefectures (>8,000 tons) [statistics from MAFF]^1^. White markers indicate northernmost and southernmost prefectures producing Japanese pear. Squares indicate locations of flowering disorder surveys.

**FIGURE 2:**
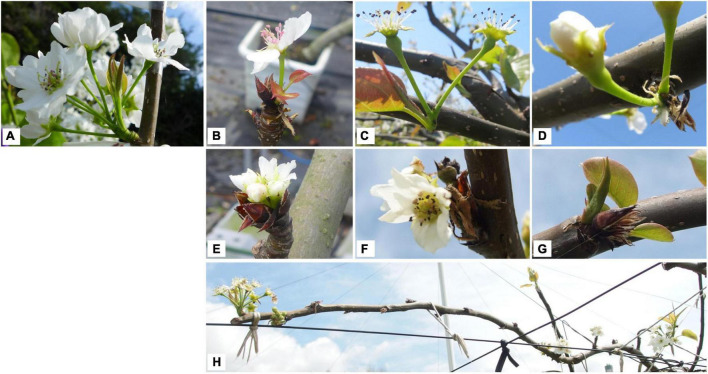
Typical symptoms of flowering disorder at blooming. **(A)** Healthy (normal) flower. Pear flower bud is a mixed flower bud, with one or two floral primordia (constituted of several florets) and sometimes leafy primordia. **(B–D)** Flowers with only one or two florets blooming. **(E,F)** Flowers with shortened peduncle. **(G)** Flowers with all florets aborted and only elongated leaf primordia. **(H)** Flower buds located at distal parts (on long or spur shoots) bloomed but those on basal parts were delayed or did not flower.

Asian pears (Oriental pears), such as Japanese pear (*Pyrus pyrifolia* Nakai), *Pyrus bretschneideri*, and *Pyrus ussuriensis* (family Rosaceae), are cultivated worldwide, but mainly in east Asian countries including Japan and China. Most areas of Japan are in the temperate or subarctic zone, but the southern islands (south of 25° north latitude) are in the tropical zone. In Japan, diverse fruit trees are cultivated to take advantage of the diverse climate. Japanese pear, a traditional deciduous fruit tree in Japan, is the third most productive fruit tree after satsuma mandarin (*Citrus unshiu*) and apple (*Malus* × *domestica*), with a production of 209,700 tons in 2019 [statistics from the Ministry of Agriculture, Forestry and Fisheries (MAFF)].^1^ In Japan, satsuma mandarin is grown mainly in warm regions and apple mainly in cold regions, while Japanese pears are cultivated nationwide from warm areas at low latitudes (31° North latitude) to cold areas at high latitudes (43° North latitude) (Figure 1 and Table 1). Since the 2000s, erratic flowering has occurred in Japanese pear trees in years with mild winters. This has been observed in trees growing in greenhouses (irrespective of artificial heating) and in warmer regions (low latitudes) and in field-grown (open-air) trees. The symptoms of this disorder include delayed blooming, flower bud abortion, reduced number of florets, smaller size of flowers and peduncles, injured or dead flower buds, lack of uniformity in bud break and blooming, lower bud break rate of both floral and vegetative buds, and ultimate bud loss, especially in the basal parts of the long (succulent) shoots (Sugiura et al., 2010; Figure 2). Flowering disorder symptoms have even been observed in greenhouse-grown trees of the ‘Kosui’ Japanese pear cultivar, which does not often suffer from cold injury, suggesting that abnormal flowering and dormancy progression caused by warm winter weather may underlie flowering disorder. Worldwide, the same phenomena have been observed in warmer regions such as New Zealand, Israel, Brazil, and South Africa (Klinac and Geddes, 1995; Nakasu et al., 1995; Erez, 2000).

**TABLE 1:** Geographical and meteorological data [statistics from JMA]*^a^* for 30 years (1991–2020) in prefectures where Japanese pear is produced (modified from Ito et al., 2018).

Prefecture	City	Latitude (N), Longitude (E)	Mean temp (°C)^z,y^	Mean precipitation (mm)^z,x^
Hokkaido	Yoichi	43° 11′ N, 140° 47′ E	8.3	1,325.2
Niigata	Niigata	37° 54′ N, 139° 16′ E	13.9	1,845.9
Fukushima	Fukushima	37° 45′ N, 140° 28′ E	13.4	1,207.0
Nagano	Nagano	36° 38′ N, 138° 11′ E	12.3	965.1
Tochigi	Utsunomiya	36° 33′ N, 139° 52′ E	14.3	1,524.7
Ibaraki	Tsukuba	36° 05′ N, 140° 04′ E	14.3	1,326.0
Chiba	Funabashi	35° 41′ N, 139° 58′ E	15.5	1,466.1
Tottori	Tottori	35° 29′ N, 134° 13′ E	15.2	1,931.3
Fukuoka	Asakura	33° 25′ N, 130° 39′ E	15.9	1,953.0
	Kumamoto	32° 46′ N, 130° 43′ E	17.2	2,007.0
Kumamoto	Uki	32° 38′ N, 130° 41′ E	–	–
	Hikawa	32° 34′ N, 130° 40′ E	–	–
Kagoshima	Kagoshima	31° 29′ N, 130° 31′ E	18.8	2,434.7

*^z^Data from the nearest weather station at each city.*

*^y^Daily mean temperature between 1991 and 2020.*

*^x^Total precipitation between 1991 and 2020.*

*^a^http://www.data.jma.go.jp/obd/stats/etrn/index.php.*

In this review, we first introduce the developmental characteristics of floral buds of Rosaceae fruit crops in relation to dormancy phase transitions. We then introduce the long-term temperature shift in Japan and the impact of warmer temperatures from autumn to spring on Japanese pear production. As the main issue, we focus on recent reports of flowering disorder in ‘Kosui’ and the putative mechanisms behind it. The obtained knowledge about how global warming is affecting fruit tree blooming provides clues as to what will happen in the future.

## Unique Reproductive Development Characteristics of Rosaceae Floral Buds During the Tree Dormancy Phase

In Rosaceae fruit trees such as apple, peach (*Prunus persica*), and Japanese pear, the flowering period between the formation of the floral meristem (i.e., structural conversion of the shoot apical meristem to the inflorescence or flower meristem) and anthesis spans several months over autumn and winter. The developmental characteristics during this reproductive phase differ from those of other temperate fruit trees such as kiwifruit (*Actinidia* spp.) and grape (*Vitis* spp.), in which visible floral initiation and differentiation appears to occur after bud break in spring.

In pome fruit trees in the Rosaceae, including Japanese pear, the tree bears floral buds at terminal and upper lateral positions of 1-year-old shoots. These are mixed buds containing inflorescence and vegetative meristems and floral primordia. Esumi et al. (2007) observed the reproductive development of floral buds at the spur position on Japanese pear ‘Hosui’ grown in Japan. Their observations of early inflorescence development indicated that floral differentiation occurs in late June to mid-July. However, within individual trees, the flower initiation period may differ among floral buds across shoots and branches, considering that floral meristem formation occurs after terminal bud set, and the timing of terminal bud set differs across shoots. Spurs cease growing in summer, while middle and long shoots continue to grow until autumn (Yang et al., 2021). Therefore, the times when growth ceases and subsequent floral initiation occur cannot be clearly defined for Japanese pear. Although the timing of floral initiation may vary among buds, the blooming time is usually uniform among buds in Rosaceae fruit trees. Therefore, from floral initiation in summer and autumn until blooming in spring, robust mechanisms allow buds to align at a certain developmental stage, thereby preventing unexpected blooming until spring, and ensuring uniform blooming in spring.

## Floral Bud Dormancy Characteristics in Rosaceae Fruit Trees

From after bud set until bud break in spring, buds are in the dormant state where their outgrowth is relatively repressed. Bud dormancy in the Rosaceae is often categorized into two different phases, based on physiologically-based definitions, endodormancy and ecodormancy (Lang, 1987). During endodormancy, bud break is repressed by unknown endogenous factors. During ecodormancy, unfavorable external conditions rather than endogenous factors repress bud break. Endodormancy is established through environmental cues, especially low temperature in the case of apple and pear (Heide and Prestrud, 2005). A genotype-dependent prolonged period of low temperature is necessary for endodormant buds to regain the potential for active bud outgrowth (chilling requirement). The depth and length of endodormancy are not evident unless the levels of bud break competency are assessed in a forcing environment. Generally, shoots or potted trees are incubated in growth-forcing conditions for certain periods, and seasonal observations of the bud break rate are conducted. Then, the relative bud break rate or days to bud break are used to estimate the depth of endodormancy. When the bud break rate under forcing conditions is over than certain percentage (often 50%), chilling requirements are supposed to be fulfilled and buds are considered to be released from endodormancy in *Prunus* (Fan et al., 2010; Fadón et al., 2020). During ecodormancy until bud break and blooming, there is a genotype-specific heat requirement (i.e., a certain amount of warm temperatures) that is required for ecodormancy release and bud break under natural conditions. Recently, changes to dormancy terminology was proposed by Considine and Considine (2016) and ecodormancy is also referred to quiescence.

Floral bud dormancy has been morphologically characterized for several fruit tree species in the Rosaceae. The onset of floral bud dormancy, also known as the rest phase or developmental arrest, occurs after inflorescence development and floral organ differentiation (Goeckeritz and Hollender, 2021; Yamane et al., 2021). The rest phase exists in *Prunus* fruit trees: its onset in floral buds occurs after all four floral whorls have differentiated. After breaking of the rest phase, microsporogenesis occurs in anthers and macrosporogenesis occurs in carpels (Julian et al., 2011; Fadón et al., 2018; Goeckeritz and Hollender, 2021; Hsiang et al., 2021b). Saito et al. (2015) reported that, in field-grown Japanese pear, the floral bud size does not change during winter, but rapidly enlarges at the end of the ecodormancy stage just before bud break. In the case of terminal floral buds on long shoots of the apple cultivar ‘Fuji’, inflorescence meristems were found to develop slowly during endodormancy. However, the developmental speed differs among cultivars with contrasting chilling requirements (Nishiyama et al., 2021). To date, inflorescence meristem development with respect to the chilling requirement is yet to be characterized in Japanese pear.

In conclusion, in the case of Rosaceae floral buds, internal inflorescence meristems and flower primordia develop continuously during when bud break is repressed, in which floral buds can mature but meiosis does not occur towards blooming progression. In other words, floral bud dormancy progresses accompanying with flower development and maturation. In this context, global climate change from autumn to spring influences both flowering and dormancy in Rosaceae fruit trees. It is still unclear whether flowering disorder of Japanese pear results from abnormal flowering or abnormal dormancy or both. Because this disorder mainly occurs in reproductive organs (flowers) but not or rarely in vegetative organs (leaves) (see Figure 2G), we hereafter refer to this physiological disorder as “flowering disorder.” However, because significant changes in floral organ formation are not the main symptoms of this disorder, it is still unclear whether it should be defined as a flowering disorder or a dormancy disorder.

## Long-Term Climatic Changes in Japanese Pear Growing Areas and the Effect on Japanese Pear Production

With recent global warming, the annual temperature in Japan has risen at a rate of 0.124°C/decade from 1898 to 2019 (Sauter et al., 1996). The forward trend in fruit tree flowering has been reported in Europe (Menzel, 2003; Chmielewski et al., 2011; Legave et al., 2013), North America (Nemani et al., 2001), and the Southern Hemisphere (Grab and Craparo, 2011; Webb et al., 2011). In Japan, it has been observed in Japanese pear (Ito and Ichinokiyama, 2005; Toya and Kawase, 2011) and apple (Fujisawa and Kobayashi, 2010; Sugiura et al., 2013).

**FIGURE 3:**
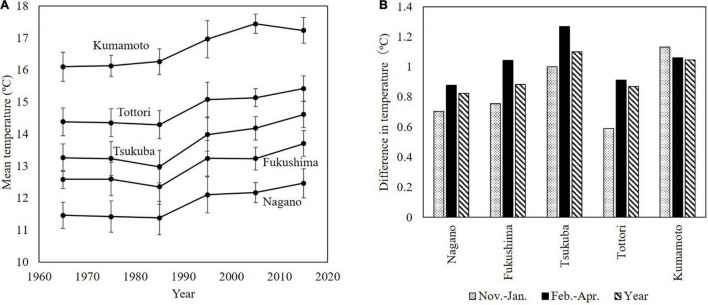
Mean temperature and temperature differences over the last 60 years. **(A)** Changes in decadal mean temperature in main production areas of Japanese pear. **(B)** Seasonal increase in mean temperature from the 30-year period in 1960–1989 to the subsequent 30-year period (1990–2019). Temperature data were recorded at Japan Meteorological Agency observatories [statistics from JMA]*^a^*.

Temperatures in Japan have risen over a long period since the end of the 19th century. In the last 50 years, this increase was particularly large in the 1990s. The changes in the decadal mean temperature in the main Japanese pear production areas did not change noticeably from the 1960s to the 1980s, but rose markedly in the 1990s and have continued to rise gradually ever since (Figure 3A).

Figure 3B shows the seasonal increase in mean temperature from the 30-year period of 1960–1989 to the subsequent 30-year period (1990–2019). In many areas, much of the period from November to January corresponds to the endodormancy period of Japanese pear, and that from February to April corresponds to ecodormancy and the blooming period. In most areas in Japan, the increase in the mean temperature from February to April has been larger than the increase in the annual mean temperature.

The most widely grown cultivar of Japanese pear in Japan is ‘Kosui’, which accounted for 39.9% of all Japanese pear production in 2018 [statistics from MAFF]^2^, followed by ‘Hosui’, ‘Niitaka’, ‘Nijisseiki’, and ‘Akiduki’. ‘Hosui’ and ‘Akiduki’ have a low chilling requirement for breaking of endodormancy, while ‘Kosui’ has mid- and ‘Niitaka’ and ‘Nijisseiki’ have high-chilling requirements (Tamura et al., 2001; Takemura, 2012). In the following paragraphs, the phenological changes of Japanese pear are described using ‘Kosui’ as the example.

Due to global warming, the flowering date of the Japanese pear is becoming earlier throughout the country. The blooming time of Japanese pear in the field in Mie Prefecture (Ito and Ichinokiyama, 2005) and Chiba prefecture (Toya and Kawase, 2011) has advanced at a rate of 3 days/decade. This is mainly due to the exponential increase in the developmental rate of buds during the ecodormancy period as the temperature rises (Sugiura et al., 1991).

**FIGURE 4:**
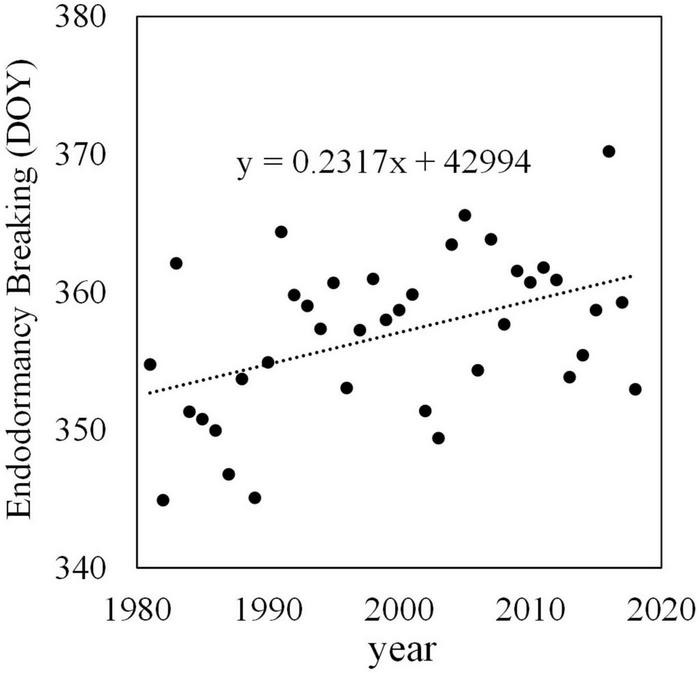
Endodormancy breaking date of Japanese pear ‘Kosui’ in Tsukuba. Endodormancy breaking date estimated from the DVR model (Sugiura and Honjo, 1997) and observed hourly temperature. DOY, day of the year (number of days from January 1).

Although there are no historical records, it is estimated that the endodormancy breaking date of Japanese pear has gradually become later. Figure 4 shows past endodormancy breaking dates of ‘Kosui’ in Tsukuba estimated by adapting observed temperatures to a development rate (DVR) model (Sugiura and Honjo, 1997). This DVR model is a chill unit model, and it was developed by subjecting potted Japanese pear trees to different temperature treatments in a growth chamber. The chilling requirement to break endodormancy of ‘Kosui’ was estimated to be 750 h below 6°C and 1,160 h at 9°C, with no break in endodormancy above 12°C. The endodormancy breaking date of ‘Kosui’ was estimated to have been delayed at a rate of 2.4 days/decade.

In Japanese pear production, flowering disorder is the main problem caused by changes in temperature during the dormancy period. Consequently, we have focused on flowering disorder in this review. However, it is noteworthy that rising temperatures during spring, corresponding to the flowering period following dormancy, also cause other major problems in Japanese pear production.

First, global warming has shortened the period of market availability nationwide. To supply the market with high-quality Japanese pears for a long period, the regional differences in harvest time are just as important as varietal differences. Because Japanese pear fruits do not require additional ripening and cannot be stored, they are shipped immediately after harvest. Because the length of the period from blooming to maturity varies little among regions, Japanese pears are shipped earlier in warmer areas where blooming occurs earlier than it does in colder areas. However, because regional differences in the blooming period have become smaller, the regional differences in the harvest period have also become smaller. The regional differences in blooming time have become smaller because rising temperatures have delayed the breaking of endodormancy with various levels depending on the regions. The long daily chilling hours in cold regions can compensate for the delayed breaking of endodormancy, so that this delay is shorter in colder regions than in warmer ones. This means that the acceleration of the blooming time is greater in colder regions than in warmer ones. Consequently, the difference in the beginning of the harvest of ‘Kosui’ between Kagoshima and Ibaraki prefectures has been reduced from about 15 days to about 8 days in the 30 years since 1987. The period when ‘Kosui’ can be shipped has become shorter in Japan, resulting in an imbalance between supply and demand.

Second, earlier bud break in the spring increases the possibility of encountering cold temperatures after blooming, and thus increases the risk of frost damage (Cannell and Smith, 1986; Murray et al., 1989; Heide, 1993; Myking and Heide, 1995). If spring temperatures increase, the last frost date should be earlier as well. However, in some areas, blooming occurs earlier than the last frost. The risk of late frost damage is higher in cold climates because the blooming date has become earlier to a greater extent in those areas.

## Main Flowering Disorder Symptoms in Field-Grown Japanese Pear Trees Are Cold Injury and Floret Abortion Caused by Warm Winters

To gain information about flowering disorder in Japanese pear, a field survey of blooming in ‘Kosui’ and ‘Niitaka’ pear trees in several orchards at different latitudes was conducted from 2011 to 2016 (Ito et al., 2018). ‘Kosui’ is the main cultivar grown in Japan. Compared with ‘Kosui’, ‘Niitaka’ has a longer chilling requirement for dormancy break: ‘Kosui’ requires 1,159 chill units (CUs) and ‘Niitaka’ requires 1,438 CUs, so they are classified as mid- and high-chill cultivars, respectively (Tamura et al., 2001; Takemura, 2012). On the basis of these observations, several possible causes of flowering disorder in pear trees subjected to warm winters were identified. Flowering disorder was pronounced only in the spring of 2016 in trees growing at lower latitudes. In the year when flowering disorder was not problematic (e.g., spring, 2015, shown in Table 2): (i) the incidence of dead flower buds was lower in ‘Kosui’ than in ‘Niitaka’; (ii) the proportion of dead flower buds on trees at Tsukuba (lat. 36°N) and Uki (lat. 32°N) was approximately 1%, which did not restrict commercial fruit production; and (iii) more flowers died on trees at Kagoshima (the lowest latitude site, lat. 31°N), resulting in approximately 3 and 10% dead flower buds on trees of ‘Kosui’ and ‘Niitaka’, respectively. The number of florets per flower bud did not differ significantly among locations for ‘Kosui’ in the 2014–2015 season, but it was significantly decreased in trees of ‘Niitaka’ growing at lower latitudes (Table 2). In contrast, more flowers died in the 2015–2016 season than in other years, with more than 3% dead flower buds at all locations for both cultivars (Table 2). The flower survival rate was much lower at Kagoshima (lat. 31°N) than at other locations (lat. 32–36°N), with approximately 30% dead flower buds in both cultivars. In addition, the number of florets per flower bud varied among locations in both cultivars; with the highest number at Tsukuba (lat. 36°N), followed by Uki (lat. 32°N) and then Kagoshima (lat. 31°N) (Table 2). The temperatures from autumn 2015 to spring, 2016 were much warmer than normal (as seen in CUs in Table 2). For both cultivars, the chilling requirement for endodormancy break was satisfied during the 2014–2015 season at all locations, but was close to being unsatisfied for ‘Niitaka’ at Kagoshima (lat. 31°N) during the 2015–2016 season (Table 2). Additionally, temperatures during the 2015–2016 season sometimes dropped suddenly, and showed large fluctuations. Between 23 and 25 January 2016, a large cold air mass advanced over the southern part of Japan, and the temperature at lower latitudes (Uki, Hikawa, and Kagoshima, lat 31–32°N) dropped abruptly. This explained the difference between the 2015–2016 season (serious flowering disorder symptoms) and the 2014-2015 season (no or light flowering disorder symptoms).

**TABLE 2:** Properties of blooming at five and three experimental sites for the Japanese pear cultivars ‘Kosui’ and ‘Niitaka’ in the 2014–15 and 2015–16 seasons (modified from Ito et al., 2018).

Year	Cultivar	Location	CU	Date of full boom^z^	Dead flower bud (%)^y^	Floret No. /flower bud^x^
2014–2015	Kosui	Tsukuba	2,791	15 April	0.0	8.1 ns ^w^
		Uki	2,324	7 April	0.8	8.2
		Hikawa	2,198	7 April	0.4	8.1
		Kagoshima	2,040	9 April	3.3	8.5
	Niitaka	Tsukuba	2,791	7 April	0.1	6.6 a
		Uki	2,324	2 April	1.4	5.4 b
		Kagoshima	2,040	1 April	8.2	3.6 c
2015–2016	Kosui	Tsukuba	2,508	12 April	3.3	8.1 a
		Tottori	2,496	16 April	8.1	8.0 a
		Uki	1,883	7 April	10.1	7.2 b
		Hikawa	1,708	7 April	7.4	7.8 a
		Kagoshima	1,473	9 April	31.6	5.4 c
	Niitaka	Tsukuba	2,508	11 April	8.5	6.7 a
		Uki	1,883	2 April	4.0	4.3 b
		Kagoshima	1,473	2 April	29.9	3.3 c

*^z^Full bloom: ≈80% of flower buds blooms.*

*^y^Flower bud number that did not flowered/total flower bud number × 100.*

*^x^Excluding non-bloomed flower bud.*

*^w^Different letters denote significant difference at 0.05 level with Tukey–Kramer test. ns denotes non-significant within a same cultivar.*

To understand the formation and development of flowering disorder symptoms, florets under scale leaves were observed regularly during the dormant season (Ito et al., 2018). There was no difference among regions in the numbers of total florets [living (no or light damage) + dead (damaged styles and stamens or entire floret was brown)]. Florets were differentiated to approximately eight in ‘Kosui’ and seven in ‘Niitaka’ at the induction of dormancy. However, abortion and death in florets or flower buds occurred close to blooming, and the numbers of flower buds and florets that actually bloomed were decreased. There were significant variations among regions in the number of aborted and dead florets, and consequently, in the flowering rate and/or the number of blooming florets per flower bud.

**FIGURE 5:**
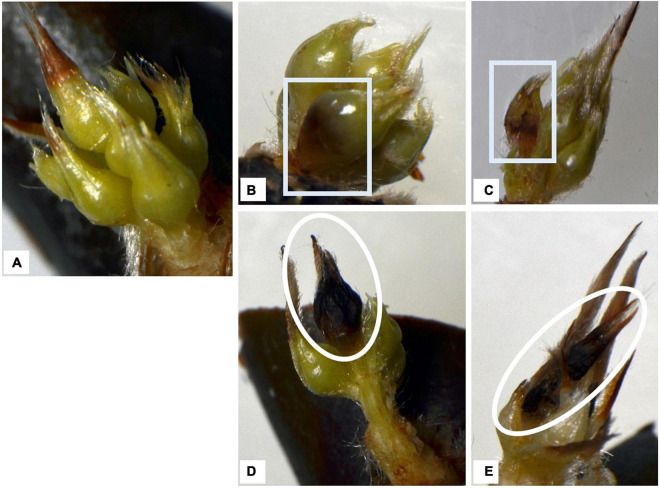
Typical appearance of flower buds of two cultivars after removing outer scale leaves. Trees were grown at five localities and observed in the 2015–2016 season. Rectangles indicate floret damage, ovals indicate floret abortion. **(A)** Healthy florets, **(B,C)** injured florets, **(D,E)** (distal) florets aborted in winter (modified from Ito et al., 2018).

The floret damage/injuries observed during dormancy could be classified into two types: (ii) “floret injury,” where the surface and/or the inside of the floret was partly or completely brown (e.g., Figures 5B,C); and (ii) “floret abortion,” where the floret was shrunken, completely brown, or had dropped from the base (e.g., Figures 5D,E). Compared with basal florets, the distally positioned florets were aborted more frequently.

**FIGURE 6:**
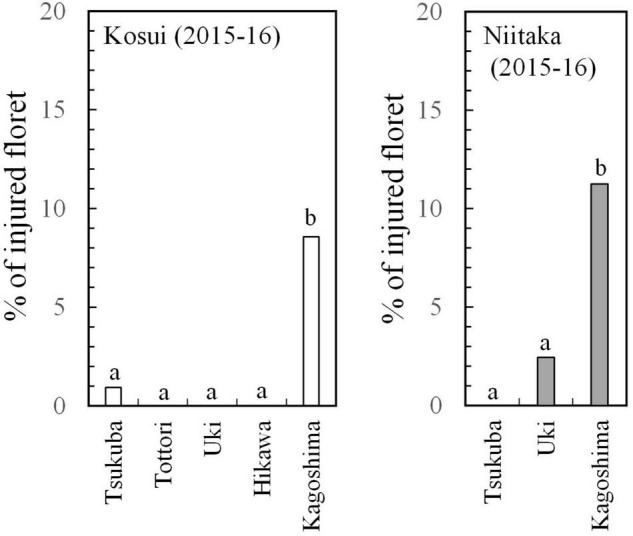
Proportion of injured florets out of total number of florets counted at blooming (observed in March 2016) (modified from Ito et al., 2018).

**FIGURE 7:**
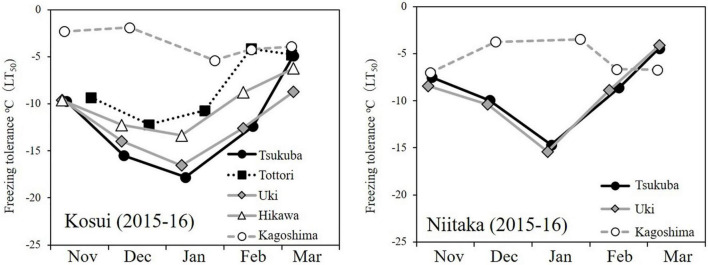
Seasonal changes in freezing tolerance of axillary flower buds of two cultivars grown at five or three localities (2015–16 season) (modified from Ito et al., 2018).

The incidence of floret injury was similar in ‘Kosui’ and ‘Niitaka’ at the same latitude (Figure 6) and showed the highest rate at Kagoshima (lat. 31°N). Floret injury was not observed in the samples collected before 27 January. A large cold mass passed over the area on 23–25 January and the temperatures dropped abruptly. Thus, any injuries observed were caused by freezing damage. The freezing tolerance [lethal temperature for 50% survival: LT_50_ (°C)] of flower buds was assessed approximately monthly during the dormant period. The seasonal patterns of flower bud freezing tolerance (LT_50_) were quite similar between ‘Kosui’ and ‘Niitaka’ at the same locations (Figure 7). At all locations except for Kagoshima, the freezing tolerance increased as the temperatures became colder and reached their maximum levels between late-December and early January. In Kagoshima, however, the freezing tolerance remained at a low level (approximately −5°C) throughout the season both in ‘Kosui’ and ‘Niitaka’, whereas the minimum air temperature on 25 January dropped to −6.6°C (lower than the LT_50_). Thus, the trees at lower latitudes had a higher risk of failing to acclimate to the upcoming freezing temperatures.

**FIGURE 8:**
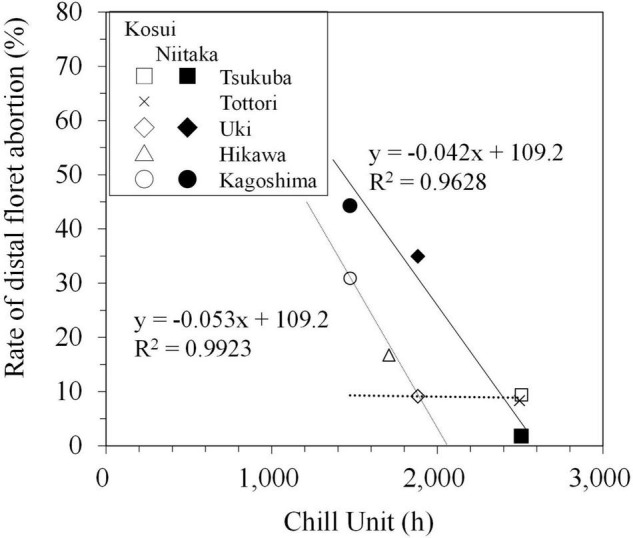
Relationship between chill units and rate of distal floret abortion in ‘Kosui’ (open symbols) and ‘Niitaka’ (closed symbols) (modified from Ito et al., 2018).

The incidence of distal floret abortion was higher in the high-chill cultivar ‘Niitaka’ than in the mid-chill cultivar ‘Kosui’. The actual number of aborted distal florets was difficult to judge, because some of them dropped from the base, so they could not be observed during regular monitoring. Therefore, the number of aborted distal florets was defined as the difference between the number of florets that bloomed and the number that actually differentiated (i.e., maximum floret number recorded during the dormant period). The rate of distal floret abortion (number of aborted florets/number of differentiated florets) was negatively correlated with CU up to approximately 1,900 in ‘Kosui’ and 2,500 in ‘Niitaka’ (Figure 8), suggesting that insufficient chilling may have caused distal floret abortion. Since Japanese pear florets differentiate from the basal to distal sites, the deficiencies in low temperature may have stronger negative impacts on distal florets because they differentiate later, thus inhibiting their subsequent development.

The degree of the influence whether freezing damage or cold shortage has a greater impact depends largely on the weather conditions of the year. For example, the winter of 2015–2016 observed to have enlarge the damage caused by freezing under large fluctuations of temperatures (especially abrupt and transient low temperatures around January 24), but similarly warm 2016–2017 had relatively small fluctuations in temperatures and consequently a small incidence of cold injury (cold injury incidence in spring, 2017: Tsukuba 2.4%; Kagoshima 0%, both ‘Kosui’ and ‘Niitaka’ included). The rates of the dead flower bud (%) were high, and more frequently observed in Kagoshima (13.2%) than in Tsukuba (0%) for ‘Kosui’, and similar in Tsukuba (7.9%) and Kagoshima (5.6%) for ‘Niitaka’.

## Flowering Disorder Occurs in Some Specific Trees Under Warm Climate Conditions and Repeatedly Occurs in the Subsequent Several Years

Flowering disorder was rarely observed in open field-grown trees after 2009, but occurred again in 2016, mainly in low-latitude areas (Ito et al., 2018). In contrast, flowering disorder in greenhouse-grown pear trees has been observed since the beginning of 2000 and continues to occur every year, with greater severity than in open field-grown trees, although there are inter-annual differences in the degree of occurrence (Fujimaru, 2004; Matsuda, 2004). In Fukuoka Prefecture (Figure 1 and Table 1), about 25% of ‘Kosui’ trees are grown in greenhouses [statistics from Fukuoka Prefecture]^3^, and flowering disorder occurs every year and has become a serious problem.

To understand the current status of flowering disorder in greenhouse-grown pear trees in commercial orchards, the occurrence of flowering disorder in trees grown in heated greenhouses was observed from 2014 to 2017 (Tominaga et al., 2019, 2021). In this cultivation system, the greenhouses are covered with plastic film from late January to early February, and the heating was set at 5°C during the night. The flowering rate (proportion of flowering buds out of total buds per tree at full bloom) was visually evaluated and scored at nine rating levels. It was judged that the lower the flowering rate, the more severe the occurrence of flowering disorder. Almost all flower buds bloomed normally in trees with a flowering rate of over 90%. Conversely, in trees with a low flowering rate, delayed flower bud break, dwarf floral organs, decreased number of florets, and flower bud abortion occurred, like in field-grown trees (Figure 2). In trees with a flowering rate of less than 30%, these symptoms were observed throughout the whole tree.

**FIGURE 9:**
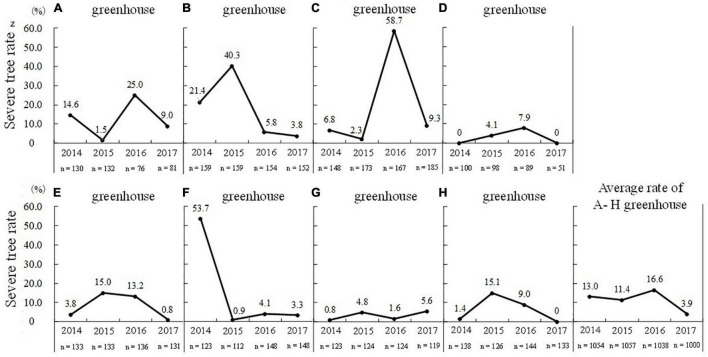
Annual trends in severe tree rate^z^ in heated greenhouses (**A–H** greenhouses) (taken from Tominaga et al., 2019 with the permission by JSHS). ^z^Trees with a flowering rate of lower than 30% were defined as “severe flowering disorder trees.” Proportion of severe trees out of all trees in the greenhouse was defined as the “severe tree rate.”

Trees with a flowering rate of lower than 30% were defined as “severe flowering disorder trees,” and the proportion of these trees out of all trees in the greenhouse was defined as the “severe tree rate.” A survey of eight greenhouses (greenhouses A–H) revealed that the “severe tree rate” varied widely from 0 to 58.7% (the maximum was in greenhouse C in the 2015–2016 season), and the rate differed among years and among greenhouses (Figure 9). The highest annual mean “severe tree rate” of 16.6% in the 2015–2016 season, which had a mild winter. In addition, flowering disorder tended to occur in the same trees, with 67.5% of trees in greenhouse A and 85.7% of trees in greenhouse B showing symptoms of flowering disorder in the three consecutive years (Tominaga et al., 2021). Thus, some specific trees in greenhouses were prone to flowering disorder, and once flowering disorder occurred in a tree, it was more likely to occur in the same tree in subsequent years.

## Lower Expression Levels of *DORMANCY-ASSOCIATED MADS-box* Genes in Trees With Flowering Disorder Suggest That Impaired Dormancy Onset May Induce Flowering Disorder

### Identification and Characterization of Potential Dormancy and Flowering Regulators in Japanese Pear

It is important to clarify the mechanisms of bud dormancy in fruit trees to better understand the mechanism of flowering disorder. Our understanding of the molecular regulators of bud dormancy–activity cycles has increased substantially in the last decade. In Rosaceae fruit trees, the potential dormancy regulators encoded by *DORMANCY-ASSOCIATED MADS-box* (*DAM*) genes, which belong to the *SHORT VEGETATIVE PHASE* (*SVP*)/*AGAMOUS LIKE 24* subfamily in the MADS-box superfamily, have been widely and extensively studied across different genera and species (Falavigna et al., 2019; Quesada-Traver et al., 2020; Hsiang et al., 2021a; Yamane et al., 2021; Yang et al., 2021). Details about the identification of dormancy-related *SVP*/*AGL24* subfamily *MADS-box* genes and the molecular regulatory network involving *DAM*s have been described in detail by Falavigna et al. (2019) and Yang et al. (2021), respectively. Although there are inconsistencies in the numbers and nomenclature of *DAM* genes identified in Asian pears (Yang et al., 2021), three *DAM* genes were first identified in the genome of ‘Kosui’ (Ubi et al., 2010; Saito et al., 2013), namely *PpyMADS13-1/2/3*. Subsequently, four *DAM*s (*PpyDAM1*–*DAM4*) were identified from Chinese pear ‘Suli’ (Liu et al., 2012; Niu et al., 2016; Yang et al., 2021). Several genetic studies have indicated the potential functions of *DAM*s in dormancy regulation. Studies on the peach *evergrowing* (*evg*) mutant strain that cannot set terminal buds indicated that the *evg* phenotype is caused by a deletion mutation in the peach *DAM1–4* genes and low or no expression of *DAM5* and *DAM6* (Bielenberg et al., 2008). This was partially supported by a functional evaluation. Transgenic poplar trees overexpressing the Japanese apricot *PmDAM6* gene, whose expression is up-regulated during dormancy induction and down-regulated during dormancy break in vegetative buds, exhibited inhibited growth and early bud set (Sasaki et al., 2011). Additionally, *PmDAM6*-overexpressing transgenic apple trees exhibited reduced growth and early bud set, and reduced bud break capability of vegetative buds during dormancy, dormancy breaking, and bud break stage (Yamane et al., 2019). In apple, *MdDAM1* and *MdDAM4* expression patterns were found to be correlated with the seasonal dormancy process, and to differ among cultivars with contrasting chilling requirements for bud break (Moser et al., 2020). Silencing of *MdDAM1* and *MdDAM4* expression eliminated terminal bud formation and dormancy induction in apple, similar to the *evg* mutant phenotype in peach (Moser et al., 2020). The overexpression of *MdDAMb* repressed bud break in apple (Wu et al., 2017). Silencing of all *SVP*s and *DAM*s in apple resulted in an *evergrowing* phenotype (Wu et al., 2021). The results of those functional evaluation studies suggest that Rosaceae *DAM*s may participate in dormancy regulation by acting as growth inhibitors and bud break repressors. In pear, virus-induced gene-silencing of *PpyDAM1*, *PpyDAM2*, and *PpyDAM4* resulted in early bud break (Gao et al., 2021). Tuan et al. (2017) suggested that *MADS13-1* (also named *PpDAM1*) is involved in dormancy regulation *via* activating abscisic acid (ABA) biosynthesis (Tuan et al., 2017). Genome-wide transcriptome studies have been conducted for Japanese pear (Nishitani et al., 2012; Bai et al., 2013; Takemura et al., 2015) and Chinese pear (Liu et al., 2012). Other than *DAM*s, proposed dormancy regulator genes in pears include *Inducer of CBF expression1* (*ICE1*), *dehydrin-responsive element binding factor* (*DREB*), *ethylene-responsive factor* (*ERF*) (Takemura et al., 2015), *GA-stimulated transcript1* (*GAST1*) (Yang et al., 2019), and and *Pyrus pyrifolia HD-zip* (*PpHB22*) (Yang et al., 2018).

Regarding the molecular regulation of flowering in fruit trees, orthologs of two well-known flowering regulators in Arabidopsis, the flowering promoter encoded by *FLOWERING LOCUS T* (*FT*) and the flowering repressor encoded by *TERMINAL FLOWER 1* (*TFL1*) (Kurokura et al., 2013), were intensively studied. In apple, a close relative of pear, two *FT* homologs *MdFT1* and *MdFT2* have been identified, and their flowering promoting roles have been confirmed by overexpression in Arabidopsis (Kotoda et al., 2010). Bai et al. (2017) identified *PpFT1a* and *PpFT2a* from Japanese pear. *PpFT2a* was found to be expressed during reproductive development after floral induction and is assumed to function in floral development. Among the *TFL1* homologs, *PpTFL1* is associated with floral induction in Japanese pear (Esumi et al., 2007; Bai et al., 2017). RNAi silencing of pear *TFL1* resulted in an early flowering phenotype in European pear (Freiman et al., 2012). *FT* and *TFL1* homologs affect dormancy and bud break in some woody species. In poplar, *FT* represses growth cessation (Böhlenius et al., 2006) and promotes bud break (Busov, 2019), while *CEN/TFL1* regulates dormancy break (Mohamed et al., 2010). Plum (*Prunus domestica*) plants overexpressing poplar *FT1* were unable to enter dormancy (Srinivasan et al., 2012). Ito et al. (2016) found that blooming time may be mediated *via* the balance between the flowering-related genes *FT* and *TFL1*, whereas bud break may be regulated by *DAM* genes. Although Japanese pear *FT* and *TFL1* are yet to be functionally evaluated in genetic studies, the results of studies on related species suggest that Japanese pear *FT* and *TFL1* may participate in the regulation of flowering and dormancy.

### *DAM* and *FT/TFL1* Expression in Field-Grown Japanese Pear Trees With Flowering Disorder

Ito et al. (2018) compared the expression of pear *DAMs* in trees growing at different five latitudes, and found that the maximum transcript levels of *DAM* in ‘Kosui’ tended to be lower at lower latitudes. In ‘Niitaka’, both the increase and decrease in *DAM* transcript levels were delayed at lower latitudes (Ito et al., 2018). In contrast, the transcript levels of *FT* (*PpFT2a*) during the blooming period were lower at lower latitudes in both cultivars, whereas those of *TFL1* (*PpTFL1-2a*) during the dormant season were higher at lower latitudes. Some studies have suggested that there is a relationship between high autumn temperatures and the delay of both dormancy progression and bud burst/blooming in woody perennials (Heide, 2003; Yamamoto et al., 2010). Malagi et al. (2015) compared the dormancy dynamics of apple buds under temperate and mild winter climate conditions, and found that cold winter temperatures were strongly correlated with both the entry into, and the depth of, dormancy. These data suggest that higher temperatures before and/or at the onset of endodormancy might decrease the degree of endodormancy in ‘Kosui’, as shown by the lower maximum transcript levels of *DAM*, and heterogenous blooming of the flower buds. Higher temperatures before endodormancy break might interrupt endodormancy progression in ‘Niitaka’, as shown by the delay of the onset of the increase and decrease in *DAM* transcript levels. Ito et al. (2018) also emphasize that the expression of *TFL1* (*PpTFL1-2a*) during the dormant period was found to be negatively correlated with the ability to bloom. We can infer that chilling may be the primary cue for endodormancy break (i.e., to allow the flower bud to break), but other supplementary cue(s) may be involved in the fine-tuning of the blooming time. The balance of *FT/TFL1* expression may have a significant role in regulating this process, but further research is required to confirm this.

### Expression Analysis of *DAM* and *FT* in Greenhouse-Grown Japanese Pear Trees With Flowering Disorder

**FIGURE 10:**
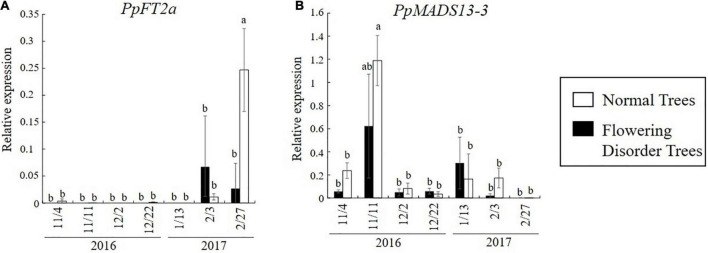
Changes in dormancy- and flowering-related gene expression in normal trees (NTs) and flowering disorder trees (FDTs) (taken from Tominaga et al., 2021 with the permission by JSHS). **(A)** Transcript levels of *PpFT2a.* **(B)** Transcript levels of *PpMADS13-3*. Relative gene transcript levels were normalized to that of *PpHistonH3*. Vertical bar indicates standard error (*n* = 3–4 axillary flower buds). Different letters indicate significant differences at 5% significance level (Tukey–Kramer test).

**FIGURE 11:**
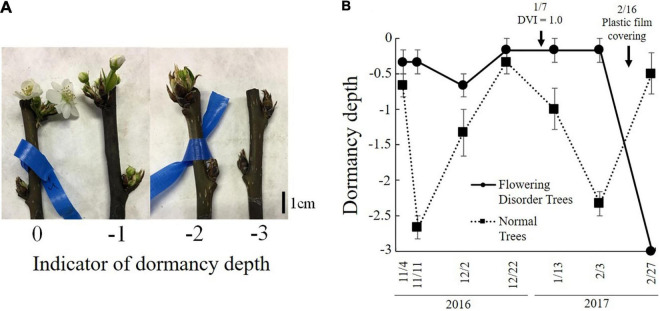
Depth of dormancy in normal trees (NTs) and flowering disorder trees (FDTs) (taken from Tominaga et al., 2021 with the permission by JSHS). **(A)** Dormancy depth and **(B)** changes in dormancy depth. Vertical bar indicates standard error (*n* = 3).

Tominaga et al. (2021) defined trees with a consecutive flowering rate of 60% or lower from 2014 to 2016 as “flowering disorder trees” (FDTs), and those with a flowering rate higher than 70% were defined as “normal trees” (NTs). These trees were studied in the 2016–2017 season. The flowering rate of FDTs in the 2016–2017 season was lower than 30% and their flowering was significantly delayed compared with that of NTs. Analyses of gene expression in the flower buds of dormant branches revealed lower transcript levels of *PpFT2a*, a flowering-related gene, in FDTs than in NTs just before flowering (February 27, 2017) (Figure 10A). Because *PpFT2a* is highly similar to apple *MdFT2*, which is involved in the process of floral organogenesis (Kotoda et al., 2010), it is possible that FDTs delayed flowering due to delayed development of floral organs. To investigate dormancy depth, cut branches were subjected to forcing conditions (3 weeks at 25°C). These analyses revealed that the lower the bud break rate, the deeper the depth of dormancy (Tominaga et al., 2021). The dormancy depths of NTs deepened on November 11 and February 3, which were considered to be the deepest stages of endo- and eco-dormancy, but that of FDTs remained shallow until February 3, before the trees were covered with plastic film, and the dormancy stage was unclear (Figure 11). During this period, the transcript level of *DAM* (*MADS13-3*), a dormancy-related gene, increased in NTs on November 11 and from January 13 to February 3, showing a negative correlation with the depth of dormancy. No such correlation was detected in the FDTs. These results suggest that flowering disorder of FDTs may be caused by abnormal dormancy progression between the endodormancy and ecodormancy periods. Future studies on the associations between abovementioned dormancy-related genes and flowering disorder will provide further clues about the dormancy status of trees with flowering disorder.

## How Do Deficiencies in Low Temperature Induce Flowering Disorder?

### Possible Relationship Between Lower Carbohydrate Metabolism and Flowering Disorder Induced by Warm Temperatures

Many studies have revealed that changes in primary and secondary metabolites in dormant buds are associated with dormancy progression. Changes in the contents of phytohormones (Liu and Sherif, 2019), sugars and carbohydrates (Tarancón et al., 2017; Zhang et al., 2018), lipid bodies (Saito et al., 2017; Grimberg et al., 2018; Veerabagu et al., 2020), and reactive oxygen species (Beauvieux et al., 2018) have been identified as dormancy-associated characteristics. Recent studies have determined the effects of chilling and chilling deprivation on the metabolomic profile of Japanese pear during dormancy progression. Ito et al. (2021) proposed that ABA levels did not coincide with endodormancy break in Japanese pear, in contrast with sweet cherry where decreased ABA levels are associated with endodormancy break (Vimont et al., 2021). It was also proposed that phytohormones regulate dormancy progression upon sensing ambient conditions, raising the possibility that the floral buds of Japanese pear are more susceptible to temperature changes than are those of sweet cherry. Several studies have determined the effects of mild winter conditions on the metabolic profile of Japanese pear. Rakngan et al. (1996) reported that cold deprivation decreased the contents of sugars and starch in shoots during the dormancy phase, resulting in less vigorous branches in the next growing season. Another study found that warm winter conditions not only affected carbohydrates, but also the fatty acid composition of lipids (Gemma, 1995). Yamamoto et al. (2010) exposed ‘Housui’ plants to 600 chilling hours below 7.2°C, less than the chilling requirement, and found that this cold deprivation treatment did not inhibit bud break but resulted in floral primordia necrosis, which was associated with low water mobility and water content (Yamamoto et al., 2010) and inhibition of carbohydrate metabolism (Horikoshi et al., 2017). The metabolic profiles of Japanese pear floral buds obtained by gas chromatography–time of flight–mass spectrometry (GC-TOF-MS) were compared between floral buds treated with constant chilling (6°C) and fluctuating chilling (6/18°C) (Horikoshi et al., 2018). Compared with constant chilling, the thermal fluctuation treatment resulted in lower levels of metabolites related to energy production. Together, the results of those studies show that warm conditions can alter the abundance of metabolites that are necessary for flower development and dormancy progression during the dormant phase. However, because sugars and carbohydrates are also associated with cold tolerance (Sauter et al., 1996), it remains unclear whether lower carbohydrate contents in warm conditions affect dormancy progression, or cold tolerance, or both. Ito et al. (2013) investigated the effects of low temperatures during winter on the sugar dynamics in Japanese pear shoots, and proposed that sugar metabolism and transport may be associated with both dormancy progression and freezing tolerance but through different mechanisms. So far, however, no metabolomics studies have been conducted on trees with flowering disorder.

### Process of Chilling Exposure, Rather Than Total Amount of Chilling, May Be Important to Ensure Precise Dormancy Progression and Robust Blooming

Field surveys have revealed that flowering disorder in field-grown Japanese pear is due to both distal floret abortion and freezing damage. The risk of flowering disorder is higher in high-chill Japanese pear cultivars than in low- or mid-chill ones, and higher at lower latitudes than at higher ones. Therefore, it is likely that warm climate conditions increase the risk of flowering disorder and also enhance the risk by interacting with the genetic regulation of the chilling requirement for the breaking of endodormancy.

In pear trees, exposure to low temperature is a prerequisite both for the induction and breaking of endodormancy (Heide and Prestrud, 2005; Takemura et al., 2011). Additionally, chilling exposure may induce floral bud maturation. For example, bulb species that are not exposed to a prolonged low-temperature period, which is necessary for normal floral organ development, produce deformed floral organs with short floral stems and abnormal petals (De Hertogh, 1974). Therefore, we propose that exposure to sufficient chilling promotes flower development by allowing dormancy to progress from endodormancy to ecodormancy, so that floral buds can develop normally. The successful progression of this process is necessary to achieve a high blooming rate and uniform flowering in spring. In contrast, the freezing tolerance of trees increases in response to shorter days, and is reinforced by low and freezing temperatures (e.g., reviewed in Junttila and Kaurin, 1990; Welling and Palva, 2006). Taken together, the results of many previous studies indicate that the higher temperatures of autumn and winter at lower latitudes may interrupt the acquisition of freezing tolerance and the progression of dormancy, resulting in a higher frequency of flowering disorder.

Considering only the chilling amounts in the 2014–2015 and 2015–2016 seasons, the theoretical chill requirement was fulfilled for ‘Kosui’ and ‘Niitaka’ at all locations. However, in experiments where potted pear trees were treated with different amounts of chilling initiated at different times and their bud break (scale leaf elongation) and flowering properties were compared, chilling provided at non-optimal times did not promote blooming (Ito et al., 2016). Thus, chilling temperatures during a certain period may allow (potential) flower buds to mature and acquire the ability to bloom, similar to the process of vernalization in cereal crops. Therefore, not only a reduced quantity, but also poor timing of the low temperature period may amplify the risk of flowering disorders. This may explain why disorders occur more frequently in floral growth than in vegetative growth (see Figure 2G). The mild winters both in recent times and in the future may retard not only dormancy progression but also flower bud maturation in perennial tree crops.

The causal factors of flowering disorder in greenhouse-grown Japanese pear trees may be not only the warmer climate, but also unknown tree factors that interfere with the response to chilling accumulation. The severity of flowering disorder depends on tree age (Klinac and Geddes, 1995), the tree training system (Mooney et al., 1992), nitrogen fertilization (Sakamoto et al., 2017), and the propensity of pear cultivars to the disorder (Klinac and Geddes, 1995; Goncalves et al., 2014). As mentioned above, a lack of chilling can lead to lower carbohydrate accumulation. This may be one of the tree factors that amplifies flowering disorder and causes it to recur in consecutive years. Tominaga et al. (2021) suggested that transient high temperatures over 20°C in winter may trigger the abnormal dormancy progression that occurs in trees with flowering disorder. In their experiments, the day with the largest difference between the mean temperature and the climatological “normal” temperature was December 22, 2016, with a maximum temperature of 20°C. Flower buds in NTs sampled on this date showed very low transcript levels of *DAM* (*MADS13-3*) (Figure 10), suggesting that exposure to temperatures over 20°C decreased the transcription of this gene. Because epigenetic regulation system may exist in Rosaceae *DAM*s transcription regulation (Ríos et al., 2014), it would be intriguing to further explore how high temperatures decrease *DAM* expression in Japanese pear dormant floral buds. High temperatures reset the chilling requirement needed to break dormancy (devernalization) in vegetables, such as radish and model plants including *Arabidopsis* (Purvis and Gregory, 1945; Bouche et al., 2015). This phenomenon has also been reported for fruit trees (Erez, 2000; Sugiura et al., 2003, 2007). Based on previous studies and the results of gene expression analyses of NTs and FDTs, the depth of endodormancy of NTs and FDTs may become shallower because of the effect of high temperatures to reset the chilling requirement.

On the basis of the knowledge gained so far, the hypothetical mechanisms of flowering disorder in greenhouse-grown Japanese pear can be summarized as follows: (1) FDTs encounter high temperatures, resulting in shallow endodormancy. (2) This leads to abnormal endodormancy progression in FDTs. (3) Consequently, farmers cover FDTs with plastic film after inappropriate dormancy progression. (4) Exposure to high temperatures due to the plastic film covering may cause flowering and bud break disorders, probably because of reduced accumulation of growth-promoting factors such as starch. (5) In the next growing season, bud break and the onset of endodormancy are delayed in trees with flowering disorder. (6) The trees that have not been exposed to chilling at the optimal time show abnormal induction and progression of dormancy. (7) As a result, these trees repeatedly show flowering disorder.

## Conclusion and Future Remarks

**FIGURE 12:**
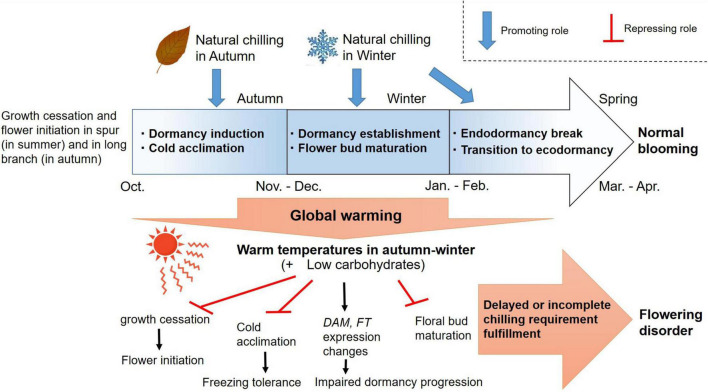
Proposed mechanism underlying flowering (dormancy) disorder in Japanese pear due to global warming.

In Japan, flowering disorder occurred in the spring of 2016 with greater severity in the high-chill Japanese pear cultivar ‘Niitaka’ than in the mid-chill cultivar ‘Kosui’, and with a higher frequency in trees located at lower latitudes (lower chilling accumulation) than in those at higher latitudes (higher chilling accumulation). The causes of this flowering disorder are attributed both to freezing injuries and to disruption of bud growth related to the shortage of chilling temperatures. Warmer autumn–winter temperatures delay the cessation of growth and interrupt the acquisition of freezing tolerance before the trees encounter midwinter freezing temperatures. Additionally, insufficient chilling hours between autumn and winter may disrupt the establishment and progression of endodormancy. Consequently, the risks of both freezing damage and endodormancy interruption may increase with increasing autumn and winter temperatures in Japan (Figure 12).

Further climate warming will increase the size of the area where flowering disorder occurs, and will increase its severity. In the long term, it is necessary to select appropriate tree species and cultivars that can adapt to the warmer temperatures predicted in the future. It also is important to continue our efforts to develop new cultivation techniques that support present productivity to mitigate the possible (catastrophic) decrease in food production in the future. The incidence of flowering disorder differs depending on the bud position within a tree. Thus, to reduce damage caused by flowering disorder, it is essential to use more robust and elastic flower buds for sustainable fruit production. Because flowering disorder occurs more frequently on the basal axillary flower buds on long shoots than on apical or distal ones, pruning methods should be adjusted to retain apical or distal flower buds (either on spurs or long shoots) rather than axillary ones. The lower frequencies of flowering disorder in apical buds than in basal buds may be related to their higher priority for growth and development (i.e., apical dominance). A full understanding of the molecular regulatory mechanisms of dormancy and flowering in Japanese pear could help to clarify the physiological and molecular basis of physiological disorder.

For greenhouse-grown Japanese pear, robust models and/or biomarkers that precisely predict the chilling requirement fulfillment dates are urgently needed. Some farmers in warm areas force trees of the early season cultivar ‘Kosui’ in plastic greenhouses so that the shipping period is earlier. Because farmers cannot visually judge whether the chilling requirement has been satisfied, flowering disorder is likely to occur if forcing conditions are applied before sufficient chilling. Flowering disorder has been observed in grape and peach in forcing cultivation. The DVR model developed in Japan provides a specific development index (DVI) value (usually DVI = 1–1.2) that serves as an index of endodormancy completion and fulfillment of the chilling requirement (Tominaga et al., 2021). However, the results of several studies suggest that the total amount of chilling exposure cannot be directly linked to the fulfillment of the chilling requirement. Rather, the process of chilling exposure during autumn to spring is more important for buds to progress through dormancy towards flowering. For example, for stone fruits produced in the United States, a dynamic model (Fishman et al., 1987) that considers not only the chilling amount but also the method of chilling is often used. This model can successfully predict the effects of chilling to fulfill the chilling requirement. Additionally, even though chilling is necessary for not only for bud break (dormancy break) but also for flower bud maturation (dormancy completion and progression), dormancy-related thermal models including the DVR model and the dynamic model are based only on bud break competency. To predict the floral bud maturation point, it will be important to monitor appropriate molecular and/or metabolic biomarkers. In peach, chilling requirement of different peach varieties were evaluated through the expression of five key genes (Leida et al., 2012). Studies on the molecular and metabolomic characteristics of Japanese pear floral buds and the responses of gene expressions and metabolites to mild winter conditions are important to identify appropriate biomarkers.

## Author Contributions

AT, AI, TS, and HY designed, wrote, and critically evaluated the manuscript. All authors contributed to the article and approved the submitted version.

## Conflict of Interest

The authors declare that the research was conducted in the absence of any commercial or financial relationships that could be construed as a potential conflict of interest.

## Publisher’s Note

All claims expressed in this article are solely those of the authors and do not necessarily represent those of their affiliated organizations, or those of the publisher, the editors and the reviewers. Any product that may be evaluated in this article, or claim that may be made by its manufacturer, is not guaranteed or endorsed by the publisher.

## Footnotes

1
https://www.maff.go.jp/e/data/stat/

2
https://www.maff.go.jp/j/tokei/kouhyou/tokusan_kazyu/index.html

3
https://www.pref.fukuoka.lg.jp/gyosei-shiryo/tokusankaju30.html
